# Transformation of Biowaste for Medical Applications: Incorporation of Biologically Derived Silver Nanoparticles as Antimicrobial Coating

**DOI:** 10.3390/antibiotics10030229

**Published:** 2021-02-24

**Authors:** Sevakumaran Vigneswari, Tan Suet May Amelia, Mohamad Hazari Hazwan, Govindan Kothandaraman Mouriya, Kesaven Bhubalan, Al-Ashraf Abdullah Amirul, Seeram Ramakrishna

**Affiliations:** 1 Faculty of Science and Marine Environment, Universiti Malaysia Terengganu, Kuala Nerus, Terengganu 21030, Malaysia; vicky@umt.edu.my (S.V.); ameliasmtan@gmail.com (T.S.M.A.); hazwanhazari@hotmail.com (M.H.H.); mouriyagk0172@gmail.com (G.K.M.); kesaven@umt.edu.my (K.B.); 2 Institute of Marine Biotechnology, Universiti Malaysia Terengganu, Kuala Nerus, Terengganu 21030, Malaysia; 3 Malaysian Institute of Pharmaceuticals and Nutraceuticals, National Institutes of Biotechnology Malaysia, Penang 11700, Malaysia; 4 School of Biological Sciences, Universiti Sains Malaysia, Minden, Penang 11800, Malaysia; 5 Centre for Chemical Biology, Universiti Sains Malaysia, Bayan Lepas, Penang 11900, Malaysia; 6 Center for Nanofibers and Nanotechnology, Department of Mechanical Engineering, National University of Singapore, Singapore 117581, Singapore

**Keywords:** silver nanoparticles, nanobiotechnology, biowaste, biomedical, antimicrobial coating

## Abstract

Nanobiotechnology has undoubtedly influenced major breakthroughs in medical sciences. Application of nanosized materials has made it possible for researchers to investigate a broad spectrum of treatments for diseases with minimally invasive procedures. Silver nanoparticles (AgNPs) have been a subject of investigation for numerous applications in agriculture, water treatment, biosensors, textiles, and the food industry as well as in the medical field, mainly due to their antimicrobial properties and nanoparticle nature. In general, AgNPs are known for their superior physical, chemical, and biological properties. The properties of AgNPs differ based on their methods of synthesis and to date, the biological method has been preferred because it is rapid, nontoxic, and can produce well-defined size and morphology under optimized conditions. Nevertheless, the common issue concerning biological or biobased production is its sustainability. Researchers have employed various strategies in addressing this shortcoming, such as recently testing agricultural biowastes such as fruit peels for the synthesis of AgNPs. The use of biowastes is definitely cost-effective and eco-friendly; moreover, it has been reported that the reduction process is simple and rapid with reasonably high yield. This review aims to address the developments in using fruit- and vegetable-based biowastes for biologically producing AgNPs to be applied as antimicrobial coatings in biomedical applications.

## 1. Introduction

Silver nanoparticles (AgNPs) have received great attention for diverse applications in nanobiotechnology research [1]. Generally, silver nanoparticles are smaller than 100 nm with 20–15,000 silver atoms [2]. Owning to their chemical stability; thermal, optical, and catalytic properties; and high conductivity, silver nanoparticles have garnered increasing attention [3]. In addition, free release of silver ions from the silver nanoparticles under certain conditions induces cell death of mammalian cells or microbial cells, meaning silver nanoparticles are broad-spectrum antimicrobial agents. Therefore, AgNPs have also become the most widely used sterilizing nanomaterials in various products, including food storage bags, refrigerator surfaces, and personal care products, as well as their use in drug delivery, biosensors, food technology, molecular tagging, textile manufacturing, antimicrobial coatings, anticancer agents, wound dressings, and cosmetic products [4].

AgNPs are unique in terms of their physical, chemical, and biological properties. Therefore, these nanoparticles have been exploited for various purposes. Various methods have been employed in the synthesis of AgNPs. Nevertheless, the conventional physical and chemical methods adapted in the synthesis of AgNPs seem to be very expensive and hazardous. However, biological preparation of AgNPs overcomes these limitations as it seems to be simple, rapid, nontoxic, dependable, green, and can produce well-defined size and morphology under optimized conditions. Further, biological synthesis produces high yield, solubility, and stability [5]. The synthesis of AgNPs involves top-down and bottom-up approaches: in the bottom-up approach, nanoparticles are synthesized using chemical and biological methods involving the self-assembly of atoms that grow into nanoparticles; meanwhile in the top-down approach, bulk materials are broken down into fine particles by size reduction.

In biological or biobased production, the important issue to be addressed is sustainability. This involves the type of resources used for the production of AgNPs, the reduction process, and the size and yield of AgNPs. The biological or green synthesis, as it is often referred to, utilises various biological systems such as bacteria, fungi, and plant extracts and small biomolecules such as vitamins and amino acids [6]. The use of fruit wastes is generally new and has been proposed as a good secondary resource for producing AgNPs [7,8]. Transformation of biowastes into value-added products is another way of effectively managing waste and achieving circular economy [9].

The current review discusses the biological synthesis of AgNPs using biowastes and the antimicrobial properties of AgNPs and their application in antimicrobial coatings. The review also looks into the research gaps on the applications of AgNPs and general health; for example, evaluation of possible risks such as genotoxicity, carcinogenicity, and toxicokinetics of AgNPs as individual compounds or when they are present as composites. Besides, it is important to consider any inevitable use of hazardous materials even during green synthesis and its effects on terms of usage and environmental safety. Nevertheless, progressing research on and the advancement of AgNPs emphasizes the prospective outlook of AgNP-related applications and opportunities to address the gaps and limitations.

## 2. Silver Nanoparticles (AgNPs)

Medical research, including AgNP-related research, has intensified due to the 2019 coronavirus disease (COVID-19), with total global cases still on the rise daily. AgNPs harbour antiviral attributes and have been reported to inhibit severe acute respiratory syndrome coronavirus 2 (SARS-CoV-2), which is still spreading due to the lack of effective antiviral measures [10]. Furthermore, the recent upsurge in AgNP-related research and the third Sustainable Development Goal, set by the United Nations to support the research and development of medicines against diseases and protect public health, also advocate the need for a summary of the existing literature and advancements in the field of medical AgNP research. The recent progress in medical AgNP research includes the development of antibiotics, drug delivery methods, hydrogels, wound dressings, nanocomposites, antioxidants, and antitumour, anticancer, antifungal, and antiparasitic agents, as well as the green synthesis of AgNPs [11,12,13,14,15,16,17,18,19]. Recent AgNP research related to nanocomposite formulation, tissue engineering, and the green synthesis of AgNPs has been relatively abundant.

Other AgNP-related progress has been observed to further elucidate the additional advancements of AgNP research. Reports have disclosed the use of plant extracts in AgNP synthesis for antimicrobial, antitumour, and anticancer drugs, wound healing, cytotoxicity, and water purification, albeit being significantly emphasised in the medical fields as antimicrobial coatings [15,16,17]. Table 1 shows the latest AgNP usages investigated in 2020, whereby most of the applications were apparently related to the medical industry as antimicrobial coatings, although the trends may have been partially encouraged by the current pandemic and third Sustainable Development Goal. Besides that, another progress in AgNP production worth noting is the advancement of AgNP composite properties using the bimetallic approach, which allows the adjustment of compositional percentage for application-specific composite properties [18,19,20]. Bimetallic studies have the potential to further develop nanocomposite research since this method has demonstrated enhanced outcomes, such as an improved antibacterial effectivity in Cu–AgNPs compared to sole Cu- or AgNPs [19]. In addition, AgNPs have also been involved in the design of several recent patents due to their attractive characteristics (Table 2).

The desirable traits of AgNPs have been reflected by the intensive and comprehensive research of the nanoparticles’ potential as an antimicrobial coating. AgNPs have been recurrently credited for their biocidal (i.e., viricidal, fungicidal, bactericidal, parasiticidal, mosquitocidal), theranostic, thermoplasmonic, antiplasmodial, catalytic, electrochemical, magnetic, optical, surface-enhanced Raman scattering, large surface-area-to-volume ratio, spatial confining, surface energy, high specific surface, and chemically stable properties [14,15,49,50,51,52,53,54,55,56]. The mentioned qualities of AgNPs have amplified their use in various applications, especially in the medical and electronic industries. For example, electrically conductive and small (1–100 nm) AgNPs can traverse nuclear and cellular membranes to trigger higher cytotoxicity with fewer implications than other drugs on the market [16]. Furthermore, AgNPs also allow versatile modification in terms of morphology and composite adjustments for desired applications [12,50]. The efficacy, toxicity, or production costs of AgNPs can be adjusted by exploiting a variety of accessible hybrid materials (e.g., silver/biopolymer nanocomposites), as well as forming them into spherical, diamond, octagonal, or thin-sheet forms [57,58,59]. For example, polymers embed nanoparticles and control nucleation while AgNPs augment the nanocomposite’s performance [60,61]. In brief, the properties of AgNPs have been broadly recognised, hence driving further research and development of this material.

Although AgNP investigation is relatively focused on surfaces and medicine, the research and development of AgNPs in the sensorics field for analytical and medical equipment are comparable. AgNPs have been investigated for target biosensors of DNA or cancer, surface-enhanced Raman scattering, molecular imaging of cancer cells, enhanced X-ray contrast, photothermal ablation of tumours, colorimetric sensing of heavy metals and ammonia for water purification, signal enhancers, and biological assays [62,63,64,65,66,67,68,69]. Biological assays can comprise considerable data requiring high-throughput screening technologies and AgNP compound semiconductors for biotagging. Other than their application in sensorics, the electrochemical and conductive traits of AgNPs enable their function as a power source or conductor. For example, AgNPs have been employed in power engineering, enhanced energy storage of batteries, intercalation material of electrical batteries, silver/polyaniline composites of microelectronic devices, and conductive inks [50,61,70,71].

Furthermore, AgNPs can also function as catalysts in biochemical and chemical reactions [65,72]. AgNPs have been reported to catalyse the oxidation of methanol to formaldehyde, ethylene to ethylene oxide, the reduction of 4-nitrophenol into 4-aminophenol, and the degradation of acridine orange and methylene blue dyes [72,73]. Additionally, AgNPs have also shown potential applications in the agricultural field. The mentioned nanoparticles can enhance plant growth while affecting the root length, shoot length, biomass, and seedling germination [74]. Accordingly, mixed reactions were observed with different plant species, age, nanoparticle concentration, and treatment duration [74,75,76,77,78,79]. Existing literature has upheld the attraction to AgNPs of multiple industries, especially surfaces and medicine. However, several routes regarding the biocidal mechanisms and chronic implications of AgNPs on living organisms are yet to be clarified. Consequently, thorough research is necessary to confront critical issues, such as the interaction of AgNPs with the biological system and natural environment.

## 3. Production of AgNPs

**Figure 1:**
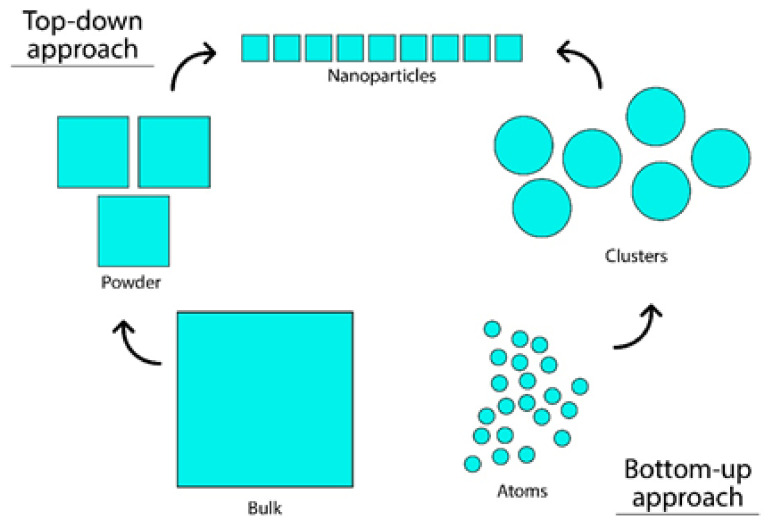
The approaches in silver nanoparticle synthesis involve bottom-up and top-down approaches.

Generally, there are two approaches involved in the synthesis of silver nanoparticles: either the “top-down” approach or the “bottom-up” approach (Figure 1), which are commonly achieved using physical, chemical, and biological methods. In the bottom-up approach, nanoparticles can be synthesized using chemical and biological methods involving the self-assembly of atoms into new nuclei, which grow into a nanoscale particle. Conversely, in the top-down approach, suitable bulk material is broken down into fine particles by size reduction with various lithographic techniques, grinding, milling, sputtering, and thermal/laser ablations [80]. In the bottom-up approach, chemical reduction is the most common scheme for the synthesis of silver nanoparticles [81,82]. Different organic and inorganic reducing agents, such as sodium borohydride (NaBH_4_), sodium citrate, ascorbate, elemental hydrogen, Tollens reagent, N,N-dimethyl formamide (DMF), and poly (ethylene glycol) block copolymers are used for the reduction of silver ions (Ag+) in aqueous or nonaqueous solutions [80,83,84,85,86,87].

The physical and chemical methods can produce well-defined AgNPs, but they have certain limitations in terms of expensive equipment usage, high energy consumption, and operation conditions, for instance, high temperature and pressure [88]. Chemical and physical methods are often costly and generate toxic byproducts; these methods involve the utilization of hazardous chemicals such as sodium borohydride (NaBH_4_) as a reducing agent, which has an adverse effect on health due to the absorption of harmful chemicals on its surface. This has brought about the development of biological synthesis, which has been recognized as an inexpensive and eco-friendly process. Biosynthesis of nanoparticles through biological approaches offers an alternative to conventional chemical and physical methods [80]. This process includes the utilization of microorganisms, plant extracts, or agricultural biowaste, which offers a green solution with limited usage of toxic chemicals [83]. It has been noted that synthesized AgNPs can have a synergic relationship with the antibiotic levofloxacin, increasing the total antimicrobial properties against Gram-positive and Gram-negative pathogens [89], as well as exerting cytotoxic effects on different cancerous and normal cell lines. In addition, AgNPs are highly efficient due to a high surface-area-to-volume ratio, which can easily disrupt and penetrate bacterial cells when compared to silver ions alone [1].

### Biological Approach of Nanoparticle Synthesis

In the green synthesis of AgNPs, plant constituents, including proteins, enzymes, and carbohydrates, are used together with AgNO_3_ to formulate nanoparticles that can easily interact with targeted biomolecules [90]. The bioactive components in plants act as reducing agents to facilitate the reduction of metallic ions to nanoparticles [91]. Several studies have reported that the biological synthesis of nanoparticles has gathered much attention due to the high-yield production of AgNPs. Furthermore, this biological approach is green, cost-effective, and biocompatible without the use of toxic chemicals. Moreover, in the biological synthesis of AgNP, it is relatively simpler to control the size, shape, and distribution of nanoparticles by optimizing several parameters, including the synthesis methods, quantity of precursors, temperature, pH, and quantity of reducing and stabilizing agents [92]. The biological synthesis of AgNPs utilises various biological systems, such as bacteria, fungi, and plant extracts, and small biomolecules such as vitamins and amino acids [6]. In this green chemistry approach, several bacteria including *Pseudomonas stutzeri* AG259, *Lactobacillus* strains, *Bacillus licheniformis*, *Escherichia coli* (*E. coli*), *Brevibacterium casei*, fungi including *Fusarium oxysporum*, and plant extracts including *Allophylus cobbe*, *Artemisia princeps*, and *Typha angustifolia* were utilized [93].

**Figure 2:**
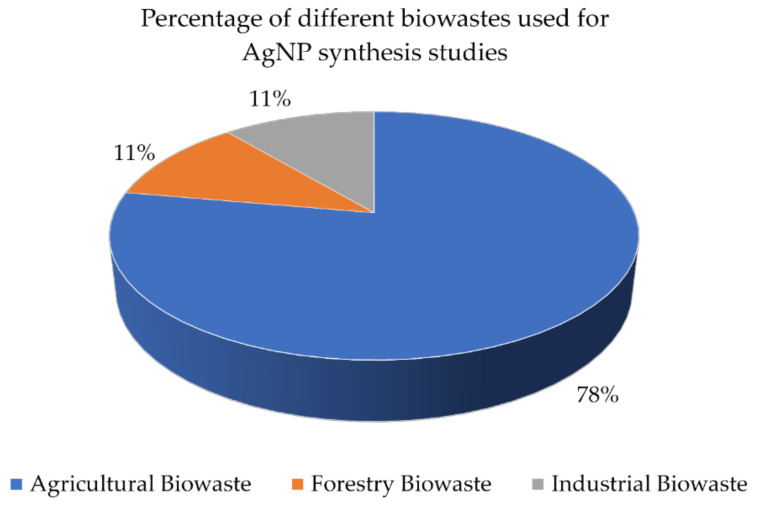
A pie chart visualizing the general dominance of prominent biowastes in the field of silver nanoparticle (AgNP) synthesis.

Biowaste is also among the various biological systems that can be used to biologically synthesise AgNPs. The consumption of biowastes to produce AgNPs employs a simple, safe, eco-friendly, cost-effective, waste-to-wealth, and circular bioeconomy approach [94,95,96]. The prominent categories of biowastes used to biologically synthesise AgNPs are forestry, industrial, and agricultural wastes (Figure 2) [95,96,97,98]. Different types of biowastes, such as forest biomass, paper industry waste, sugar industry waste, spent coffee grounds, husks, as well as vegetable and fruit peels, have been broadly researched for the production of AgNPs (Table 3). Existing literature has shown a focus of research attention on AgNP synthesis using fruit pericarp, likely due to the ease of access, relatively lower cost, and abundance of this waste type. Biowastes are sustainable renewable energy sources, hence the management and utilization of biowastes can help to alleviate the global energy consumption demand [9]. Biowaste-derived AgNPs are thus not only of low economic value but also environmentally and economically sustainable [94]. The efforts in the biological conversion of waste to a valuable product, AgNPs, not only reduce waste generation and AgNP production costs, but also help to shift the economy from a cradle-to-grave to a cradle-to-cradle concept [9]. Therefore, the biosynthesis of AgNPs from biowastes is considered to reinforce the valuable utilization of biomass for the synthesis of metal nanoparticles. Consequently, the major advantage of biological synthesis is the elimination of toxic chemicals, and the use of biological molecules for the synthesis of AgNPs is profoundly eco-friendly and pollution-free.

## 4. Antimicrobial Properties of AgNPs

In the context of antibacterial purposes, silver metal has been a favoured material even 2000 years ago, and especially starting from 19th-century civilizations [117]. The studies of silver as a material that exhibits antimicrobial properties have also gained increasing attention and extensive experimentation among researchers, owing to its range of bactericidal attributes, potencies, low level of toxicity, and numerous utilisations as a sanitizer [118]. Silver nanoparticles (AgNPs) especially have prevailed in showing positive results in inhibiting the bacterial growth of a variety of Gram-negative and Gram-positive bacteria [119]. As many as 650+ microbes that are known to be affected by the antimicrobial activity of silver-based compounds have been recorded [80,120].

The usage of AgNPs has its own precedence of having lower reactivity compared to silver ions, making this material greatly applicable for medical and restorative purposes [121,122,123]. Out of the wide range of possible implementations of AgNPs in these fields, numerous research and development projects have been extensively deployed towards their auspicious utilization in wound dressings, tissue scaffolds, and protective clothing use, among others [124,125,126]. Furthermore, licensing bodies such as the FDA and EPA of the USA, the SIAA of Japan, the Korea Testing and Research for Chemical Industry, and the FITI Testing & Research Institute of Korea have granted the development of numerous products containing or associated with AgNPs [127]. This demonstrates how AgNPs efficacy as antimicrobial material has been taken advantage of in being implemented domestically and clinically [120].

### 4.1. Mechanism of Action of AgNPs as Antimicrobial Agent

As a type of metal, AgNPs possess an oligodynamic effect, which is related to the biocidal effects of metals, particularly heavy metals, that may potentially take place in low concentrations of that particular material [118]. There are some important facets regarding the particular antimicrobial features of AgNPs and their innate physical and chemical attributes, which sustain their nanoscale size, enhance their rapid distribution, and avoid accruement [128]. The large surface areas of AgNPs are the important feature of their enhanced oligodynamic effect in their capability to cohere with bacterial plasma membranes, their aptitude for cell perforation, to induce synthesis of reactive oxygen species (ROS) and free radicals, and to function as signal transduction pathway modulators of microorganisms [129]. In addition, the distinctions in AgNPs’ physicochemical traits, such as size [130], morphology [131], oxidation and dissolution states [132], and charges and coating of surfaces [133,134], have great impact on their antimicrobial activity [135,136].

### 4.2. Antibacterial Effects of AgNPs

Silver nanoparticles are becoming a hot topic among those in the scientific domain due to their wide range of bactericidal and fungicidal qualities. The effect of microbial inhibition can even affect both Gram-positive and Gram-negative bacteria, with high antibacterial activity. Currently, the mechanism of actions of AgNPs as antimicrobial agents consist of four main actions [118,123]:

1. Adherence onto the cell membrane of microorganisms.
2. Perforation of cells by AgNPs, interrupting cell molecules and causing intracellular destruction.
3. Effecting toxicity of microbial cells through the synthesis of ROS that stimulates cell oxidative stress.
4. Obstruction of cell signal transduction pathways.

**Figure 3:**
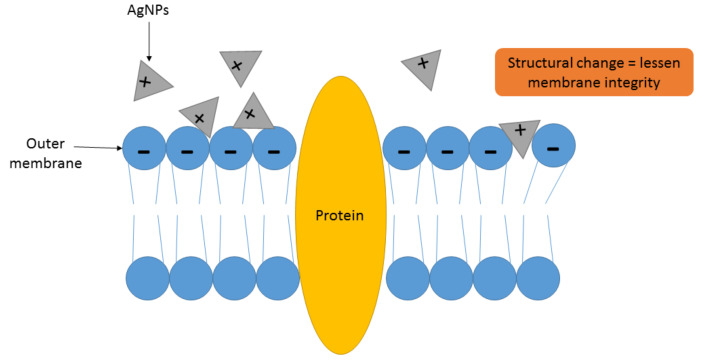
The mechanism of action of AgNPs affected through cell binding via the mechanism of AgNPs’ adhesion to the cell membrane.

In a condition where AgNPs are introduced to any microorganism, the positive charge of silver ions prompted from the oxidation of AgNPs will be attracted towards the negatively charged cell membrane of microbes due to the electrostatic effect, causing the nanoparticles to fasten to the cell wall or membrane [137]. The changes in the shape of cell membrane are permanent and irreparable when AgNPs are completely attached to the plasma membrane of the microorganism, which can cause impairment of the lipid bilayer and permeability of the plasma membrane and thus ability to enclose its content of cytoplasm and nucleoplasm [138]. The modifications in the morphology of the cell can result in affecting the cell’s capability to separate the cell from the surrounding interstitial fluid. As an example, formation of silver ions by the nanoparticles will reorient the movement of potassium ions (K+) from the inside and outside of the cell, which can impede the mobilization activity of essential materials of the cell [118]. Moreover, increment of membrane permeability may induce the depletion or discharge of intracellular contents including cytoplasm, ions, adenosine triphosphate (ATP), and proteins, which can engender the formation of a ghost cell of a microbe. Excretion of all essential materials out of the microorganism through membrane leakages, leaving behind an empty microbial cell envelope, is known as the ghost cell effect [139]. A study by Vazquez-Munoz et al. proved that the coherence of the plasma membrane of Gram-negative bacteria (*E. coli* and *Salmonella typhirium*) was disrupted by AgNPs’ bactericidal activity through the action of membrane depolarization and destabilization, which was observed via transmission electron microscopic (TEM) images. Figure 3 shows the mechanism of AgNPs’ adhesion to the cell membrane [140].

**Figure 4:**
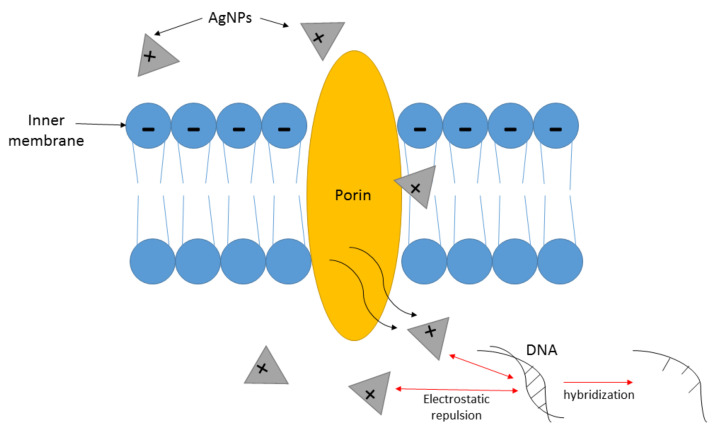
The mechanism of action of AgNPs affected through membrane penetration assisted by electrostatic repulsion and hybridization.

Besides the mechanism of binding onto the surface of the membrane, AgNPs can also pierce through the microbes and cause disturbance of crucial biomolecules and cellular activity [118]. There are water-filled channels known as porins located in the extracellular membrane of Gram-negative bacteria (e.g., *E. coli*), which can be a passageway for AgNPs to invade the intracellular environment. Following successful AgNP invasion, they will begin to cohere with biomolecules and cellular structures, including proteins, DNA, lipids, and mitochondria, that can deteriorate the inner composition of the bacteria. In addition, the free-roaming silver ions discharged from AgNPs will cohere with negatively charged proteins, resulting in modifications of protein composition and ultimately protein degeneration [118]. There is a study that demonstrated the inhibition activity of AgNPs by hindering the respiratory chain dehydrogenase through the metamorphosis of a number of enzymes, such as glycerol-3-phosphate dehydrogenase into dihydroxyacetone, in a Gram-positive bacterium (*S. aureus*), which concluded with the obstruction of the bacteria’s normal growth and metabolic activity [141]. AgNPs can also trigger DNA denaturation and impede the cellular growth of bacteria when in contact with bacterial DNA [142]. Additionally, the stability of DNA composition can be lessened through AgNP–DNA interactions due to the occurrence of electrostatic repulsion between these two because of their similar polar charge [143]. Moreover, detachment of double-stranded DNA into single strands through a hybridization process may occur due to interfaces between silver ions and DNA, causing dissociation of H-bonds in DNA strands [144]. The simplified mechanism is illustrated in Figure 4.

**Figure 5:**
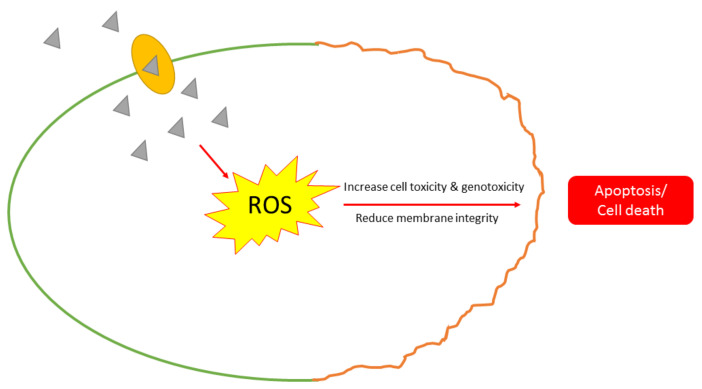
The mechanism of action of AgNPs affected through the formation of reactive oxygen species (ROS).

Reactive oxygen species (ROS), a type of cellular oxidative stress of microorganisms, is another course of action affected by AgNPs’ inhibiting activity [145]. AgNPs can cause an increment of cell oxidative stress due to the nanoparticles’ capability of generating ROS and free radicals [118]. The potential of nanoparticles to prompt lipid destruction, perforation of biomolecules, and also programmed cell death are things to look into as it suggests the occurrence and generation of intercellular ROS inside the cells is one of the biggest factors to determine the toxicity of AgNPs [146]. Su et al. reported that treatment with clay and AgNPs could enhance the bactericidal properties towards *S. aureus*, *P. aeruginosa*, and *Streptococcus pyrogens*, causing a declination of membrane integrity in response to the formation of ROS, followed by cell death [147]. There is another study that proposed that the antibacterial activity due to ROS generation is dependent on the size of AgNPs, where nanoparticles with a concentration of 10 mg/L resulted in higher levels of ROS production in *Azotobacter vinelandii* and *Nitrosomonas europaea* compared to 50 mg/L of AgNPs [148]. The method of ROS formation in bacterial cells induced by AgNPs is shown in Figure 5.

**Figure 6:**
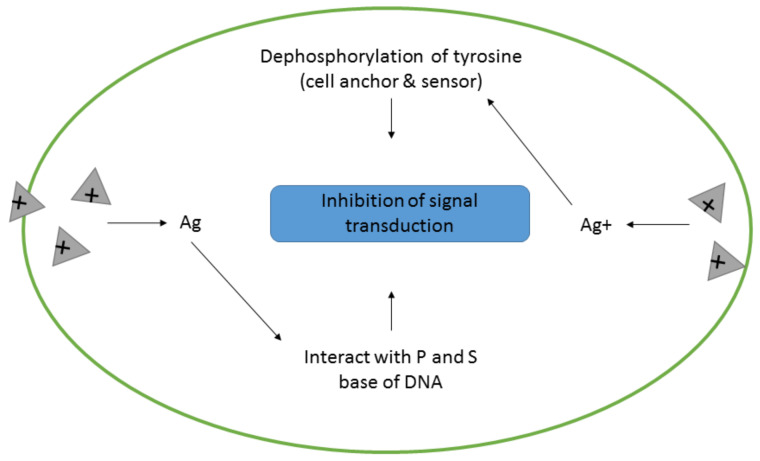
The mechanism of action of AgNPs effected through the inhibition of signal transduction [117].

The mechanism of action involving pathway signaling is influenced by the phosphorylation and dephosphorylation cascade of important enzymes or protein contents that is required for growth of bacteria and cellular activity [118]. The distinctive physicochemical traits of AgNPs may bring about the potential for another mechanism of bacterial inhibition through functionality of these nanoparticles as modulators of cell signal transduction [149]. A study had experimented on the capability of gold–silver nanoparticles to effectuate bacterial cell apoptosis through destruction of the bacterial actin cytoskeletal network [126]. The study also discovered that the cytoskeletal actin MreB (an actin homologue), which has a crucial responsibility in the modulation of local cell shapes and survivability, was experiencing changes in morphology, causing an increase of membrane liquidity and thus mediating cell destruction. Figure 6 shows the mechanism of action of bacterial inhibition through pathway signaling modification.

Different strains will experience different mechanisms of action of nanoparticles’ antibacterial activities. Table 4 lists the microbial inhibition studies of AgNPs, including their mechanism of action.

## 5. Applications of AgNPs in Antimicrobial Coatings

**Figure 7:**
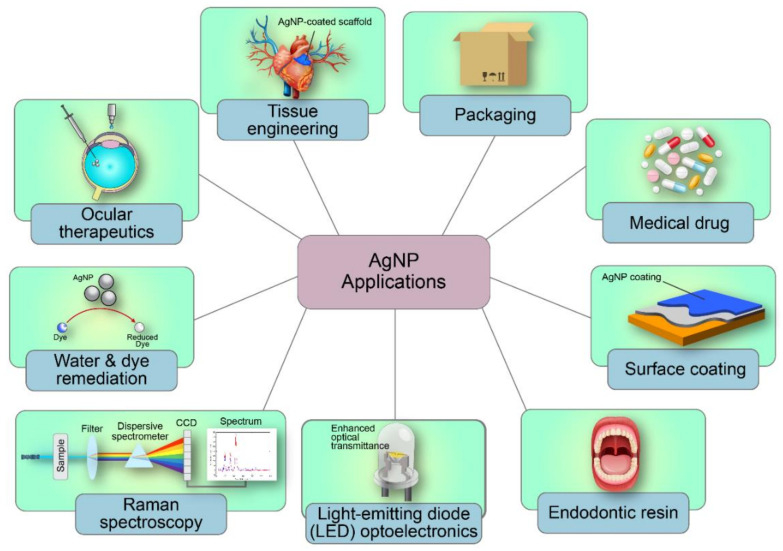
The various applications of AgNPs.

AgNPs have been investigated for a multitude of applications due to their qualities. The existing commercial products that employ the use of AgNPs include surfaces, textiles, food containers, nutrient supplements, cosmetics, packaging materials, electronics, domestic appliances, water and air disinfectants, as well as medical and laboratory instruments [11,160] (Figure 7). Furthermore, AgNP research and development has also contributed to the surfaces, medical, electronic, remediation, sanitation, catalyst, and agricultural industries, as well as analytical and medical equipment. Existing literature has shown relatively abundant publications of AgNPs in the industrial fields of surfaces and medicine. The properties of AgNPs (e.g., biocidal, superhydrophobic, thermal conductive) have advocated its use as a sheet or layer to improve apparel, footwear, paint, wound dressings, cosmetics, appliances, packaging, and plastics applications [161]. For example, biocidal AgNPs allow the production of antimicrobial paints and sterile wound dressings for accelerated wound healing, which incites the option of future AgNP deployment in odourless fabrics for sensitive skin [44]. In addition, silver/polymer composites allow applications that require high thermal conductivity, such as electronic packaging, encapsulations, light-based sensors, and satellite devices [50].

The prospects of AgNPs as an accommodating surface have also promoted its application as a coating in the field of medicine. The previously mentioned qualities of AgNPs have fostered its application as antimicrobial medical coatings in scaffolds, skin recipient and donor sites, wound dressings, clinical fabrics, cancer therapy, protein detection, antibiofilm catheters, hydro- or aerogels, and gene or drug delivery. The diverse applications of AgNPs in medical coatings demonstrate the prominence of the AgNPs’ superior antimicrobial trait. For example, biocidal fabrics are essential clinical accessories since microbial pathogens can survive on laundered clothes for up to 3 months, posing infection risks to surgical wounds, bedding, or patient clothing. Moreover, infections contracted post-injury or surgery can be fatal. Besides that, AgNPs have also shown to inhibit cancer cell lines, multidrug-resistant bacterial strains, plus the herpes simplex virus type 1 (HSV-1) and influenza A virus subtype H1N1 [162,163,164,165]. In addition, therapeutic gel comprising AgNPs has shown potential to accelerate wound healing without scarring. Also, AgNP-based antimicrobial coatings have been applied in the sanitation sector for various public and domestic-use surfaces including wood, glass, and polystyrene. In conclusion, AgNPs possess immense potential as a coating enhancer for various industries, although they are remarkably valuable in the medical field due to their biocidal feature.

## 6. Future Outlook and Conclusions

The properties of AgNPs have been widely acknowledged, thus propelling the research and development of this material. The main advantages of biomediated AgNPs are their nontoxic and eco-friendly production route and properties, which reduce undesirable negative implications on human health [90]. The biologically mediated AgNP synthesis is thus safer for medical applications and essential antimicrobial coatings that are required in clinical settings. The biological synthesis of AgNPs has also been reported to be rapid and of high yield. Moreover, unlike physical and chemical methods, the biomediated synthesis of AgNPs does not require expensive equipment, high pressure, high temperature, or chemical additives (e.g., stabilizers) [80,81,82,83,84,85,86,87]. However, although the chemical synthesis of AgNP generates relatively uniform structures, the capability to produce homogenous surfaces and crystallographic structures is a current and major challenge for the biological synthesis of AgNPs. The inconsistent composition of biowaste in each batch can cause yield fluctuations and problems with standardized quality demands of the AgNP-based final product [91]. Furthermore, the availability of biowaste for large-scale continuous manufacturing and the feasibility (e.g., costs) of biowaste refining remains questionable.

**Figure 8:**
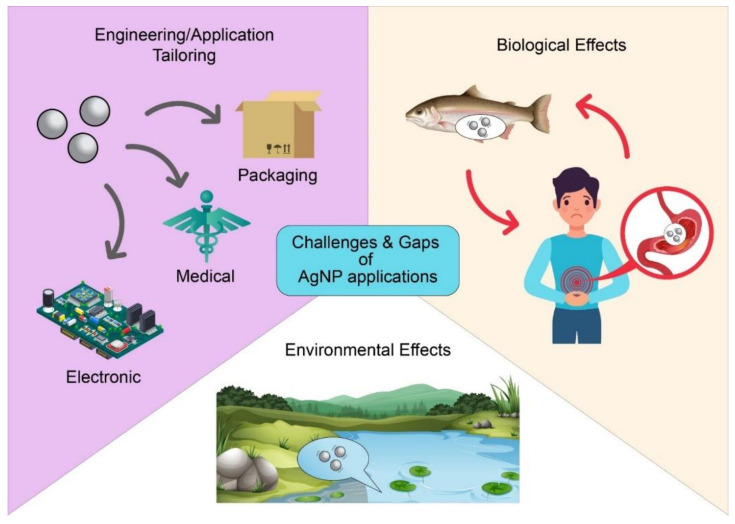
The challenges and gaps of AgNP applications.

Nevertheless, the existing and projected gaps of AgNP research should be identified concurrently to better protect public health in the direction of the third Sustainable Development Goal (Figure 8). The potential harm of loosely attached or detached nanoparticles’ entry into the biological system is still being validated. The main target organs (e.g., liver, spleen, gastrointestinal tract, lung) likely to be exposed to nanoparticles after intravenous administration should be monitored, as the mononuclear phagocytic system may also recognise these nanoparticles as foreign agents and then remove them from the circulation system [166]. Additionally, past research has been focused on the fine manipulation of the crystallinity, shape, size, and stability of AgNPs to achieve various physicochemical characteristics. Although green biological synthesis methods at present are mostly preferred over chemical and physical techniques (e.g., photochemical, electrochemical, thermal, radiation, lithographic, and laser ablation processes) to tailor nanoparticle qualities toward specific applications, any inevitable use of hazardous materials during the synthesis or structuring of AgNPs should not be overlooked in terms of usage and environmental safety [167,168,169,170]. Other gaps in AgNP research should also be pinpointed to attain more comprehensive insight in this field. Currently, limited information is available on the possible risks of AgNP exposure or lack thereof. Other knowledge gaps within AgNP research include the genotoxicity, carcinogenicity, and toxicokinetics of AgNPs as individual compounds and composites in a wide range of forms and intensities [11,171,172]. Nevertheless, as a final point, AgNP advancements continue to move forward despite the limitations and gaps confronted in this field, thus emphasising the prospective outlook of AgNP-related applications.

In conclusion, AgNP nanocomposite research has been extensively reported on and developed over the past decade. The present review reports the industrial use of AgNPs, especially in the field of medicine, chiefly due to their antimicrobial properties. Along with other attractive properties of AgNPs, research and development involving the utilisation of this material have been increasing, particularly in 2019 and 2020. Our review reveals the diverse applications of AgNP that include but are not limited to medical antimicrobial coatings. The possibility of composition and chemical modifications to achieve desirable properties and functionalities has been and is still being explored as a high-potential research area. Moreover, several studies have accentuated the need to better understand the environmental and biological impacts of AgNPs. Substantial reductions in environmental implications can be obtained with the appropriate waste management strategies of AgNP development prior to further commercialization. Furthermore, future research is also expected to focus on cost-effective processes and more sustainable materials. In addition, efforts are also observed in AgNP development for the large industrial scale of the material sciences and manufacturing fields.

## Figures and Tables

**Table 1:** Applications related to silver nanoparticles that have recently been investigated in multiple industries; a brief summary to illustrate the recent trends and advancements in the research of silver nanoparticle applications.

Industry	Application	Properties	References
Medical	Scaffold	Cyto-compatible and antibacterial scaffold	[21]
	Aerogel	Antibacterial aerogel	[22]
	Endodontics	Antibacterial Gutta-percha	[23]
	Dental acrylic resin	Antimicrobial and biocompatible resin	[24,25,26]
	Ocular therapeutics	Antiangiogenic and antibacterial therapeutic nanoparticle	[27]
	Urinary catheters	Antibiofilm urinary catheter	[28]
	Medical dressing	Wound-healing cotton fabric	[29]
Electronics	Supercapacitor and electrochemical sensing	High electrochemical capacity in 3D-printed microfluidic device	[30]
	Optoelectronic applications	Enhanced optical transmittance	[31]
	Sensing and biosensing	Stable and rapid photo-optical sensor	[32,33]
Packaging	Paper coating	Extends food shelf-life	[34]
	Film	Antioxidant and antibacterial film	[35]
Surface coating	Ceramic glaze	Antibacterial ceramic surface	[36]
Remediation	Dye degradation	Catalytic ability on dye	[31,37]

**Table 2:** Recent patents related to silver nanoparticles.

Application	Patent Title	Patent ID	References
Antimicrobial agent for wound healing	Composition comprising amino acid polymers and a bioactive agent and method of preparing thereof	US20200368176A1	[38]
Scaffold	Coating scaffolds	AU2020250274A1	[39]
Electromagnetic shielding agent	Microcellular foamed HIPS electromagnetic shielding material and preparation method and application thereof	CN111961305A	[40]
Slow-release antibacterial agent	Preparation method of nano-silver slow-release antibacterial agent	CN111973794A	[41]
Biocidal agent	Mobile device for cleaning and disinfecting room air that can be operated using a temperature difference	DE202020105700U1	[42]
Antioxidative agent	Skin cleanser	AU2020227091A1	[43]
Antimicrobial agent	Synthetic fiber with semi-permanent antibacterial and anti-fungal properties and uses thereof	KR102163245B1	[44]
Antimicrobial coating	A system that provides local cooling to the brain and spinal cord	JP2020171791A	[45]
Reducing agent in biopolymer microgel	Method of reducing an organic pollutant in contaminated water	US10793684B1	[46]
Slow-release bactericidal agent	Medicine for treating ant bite and its medicine applying plaster	CN111671844A	[47]
Colorimetric sensing of trypsin	Trypsin detection film, preparation method and application thereof and trypsin detection kit	CN111808916A	[48]

HIPS: high impact polystyrene.

**Table 3:** The various biowastes used in the green biological synthesis of silver nanoparticles.

Source	Size of the Nanoparticle	Inhibited Microbes	Application	References
*Musa* (banana peels)	50 nm	*S. aureus* *B. subtilis* *P. aeruginosa* *E. coli* *C. albicans*	Possess good antimicrobial activity against foodborne microorganisms	[99]
*Punica granatum* (pomegranate peels)	10–30 nm		Potential application in the biomedical field	[7]
*Citrus* x *sinensis* (orange peels)	10 nm	-	Prospective method of citrus canker control using orange waste	[8]
*Citrus grandis* (pomelo)	20–30 nm	-	These nanoparticles can be used as a reducing agent	[100]
*Citrus* x *limon* (lemon) *Citrus* x *sinensis* (orange) *Citrus limetta* (Mosambi peels)	9–46 nm	*E. coli* *S. aureus*	Viable resource for antioxidant extraction; anticancer properties	[80]
*Vitis* (grapes), *Carica papaya* (papaya) *Citrullus lanatus* (watermelon)	50 nm	*B. subtilis* *S. aureus* *E. coli* *P. aeruginosa*	Active food packaging	[101]
*Musa* (banana peels)			Reducing and stabilizing agent	[102]
*Punica granatum* (pomegranate)	5–10 nm	-	Offers a valuable contribution to green synthesis without adding different physical and chemical steps	[103]
*Ananas comusus* (pineapple)	9 nm	*E. faecium* *L. monocytogens* *B. cereus* *S. aureus*	Various biomedical applications, such as management of serious diseases such as diabetes and cancer	[104]
*Citrus maxima* (pomelo)	2.5–5.7 nm		Useful in extracellular synthesis of silver nanoparticles	[105]
*Punica granatum* (pomegranate), *Citrus* x *sinensis* (orange peels)	94.5 nm (orange) 74.9 nm (pomegranate)	-	Antimicrobial and wound healing	[106]
*Vitis vinifera* L. (grape pomace), *Citrus* x *sinensis* (orange residues)	90 nm (grape pomace) 96 nm (orange)	*E. coli* *S. aureus* *P. arginosa*	Reduced silver ions acting as capping agents, and also shows highest antimicrobial activity	[107]
*Citrus* x *sinensis* (orange peels)	56.1 nm	-	Paves the way for future studies on AgNP toxicity	[108]
*Punica granatum* (Saudi pomegranate fruit)	34–50 nm	*S. aureus* *S. typhi* *P. aeruginosa* *E. coli* *S. epidermidis* *K. pneumoniae*	An ideal prerequisite for efficient drug delivery	[88]
*Eucalyptus* *camaldulensis* (river red gum bark)	468.7 nm	*-*	Commercial skincare formulations	[109]
*Rhododendron ponticumu* (common rhododendron leaf waste)	10–21 nm	*L. innocua* *B. subtilis* *E. aerogenes* *E. coli*	Antibacterial ointment; textile or fabric	[94]
Spent coffee grounds	34.6–54.2 nm	*-*	Water treatment applications	[95]
*Eucalyptus* sp. (prehydrolysis waste liquor of wood)	20 nm	*P. aeruginosa* *S. aureus* *E. coli* *C. oxysporum* *P. chrysogenum* *C. albicans* *A. niger*	Biomedical applications	[96]
*Poa annua* (annual meadow grass leaf)	36.66 nm	*-*	Potential drug carrier and therapeutic	[97]
*Saccharum* sp. (sugar cane bagasse)	6–36 nm	*E. coli* *P. aeruginosa* *S. aureus*	Bactericidal applications without AgCl formation	[98]
*Allium cepa* L. (red onion peels)	14 nm	*-*	Medical and agricultural applications	[110]
*Nypa fruticans* (waste husks of Nipa palm)	10–15 nm	*B. cereus*	General range of AgNP-related applications	[111]
*Physalis peruviana* L. (outer accrescent fruiting calyx of Cape gooseberry)	25–55 nm	*E. coli* *S. typhimurium*	Antibacterial material, coating, cosmeceutical, and biomedical applications	[112]
*Cocos nucifera* L. (outer shell fibre of coconut)	-	*E. coli* *E. feacium* *P. acnes* *L. monocytogenes* *C. albicans*	Biomedical, food, and pharmaceutical applications	[113]
*Citrullus lanatus* (rinds of watermelon)	20–260 nm	*S. aureus* *E. coli* *B. cereus* *L. monocytogenes* *S. typhimurium*	Agricultural, biomedical, cosmeceutical, and pharmaceutical applications	[114]
*Solanum tuberosum* (potato peels)	20–40 nm	*-*	General range of AgNP-related applications	[115]
*Oryza sativa* japonica (rice husks)	<47.90 nm	*-*	General range of AgNP-related applications	[116]

**Table 4:** The antimicrobial activities of AgNPs [118,123].

Bacterial Strains	Size of AgNPs	Mechanism of Antimicrobial Activity	Reference
Gram-positive			
Multidrug-resistant *Staphylococcus aureus* (MMC-20)	18 ± 3 nm	Obstruction of membrane due to ROS formation	[150]
*Staphylococcus aureus* ATCC25923	3.91 nm/2.29 nm/1.59 nm	Obstruction of membrane due to ROS formation	[151]
*Staphylococcus aureus*	<100 nm	Oxidative stress caused by modification of kynurenine protein	[152]
*Bacillus subtilis*	<100 nm	Modification of kynurenine protein-mediated kynurenine pathways that inhibited growth	[152]
*Listeria monocytogenes*	23 ± 2 nm	Increase in ROS levels	[153]
*Clostridium diphteria*	28.42 nm	Cell wall hostility, denaturation of proteins	[154]
Gram-negative			
*Escherichia coli*	<100 nm	Oxidative stress caused by modification of kynurenine protein	[155]
*Escherichia coli AB1157*	8.3 ± 1.9 nm	Destruction of DNA	[156]
*Escherichia coli ATCC25922*	3.91 nm/2.29 nm/1.59 nm	Obstruction of membrane due to increased ROS formation	[151]
*Pseudomonas aeruginosa*	45 nm	Binding of AgNPs to cell wall and synthesis of ROS	[157]
*Klebsiella pneumoniae*	<100 nm	Modification of kynurenine protein-mediated kynurenine pathways that inhibited growth	[152]
*Proteus sp.*	38 nm	Estrangement of cell membrane and hindered DNA replication	[155]
*Vibrio cholera*	<50 nm	Impeded metabolic pathways	[158]
*Salmonella thyphii*	2–23 nm	Cell wall rupture	[159]

## Data Availability

The data presented in this study are openly available.
